# LiteGaze: Neural architecture search for efficient gaze estimation

**DOI:** 10.1371/journal.pone.0284814

**Published:** 2023-05-01

**Authors:** Xinwei Guo, Yong Wu, Jingjing Miao, Yang Chen

**Affiliations:** 1 School of Mechanical Engineering, University of Science and Technology Beijing, Beijing, China; 2 College of Computer Science and Software Engineering, Shenzhen University, Shenzhen, China; 3 The 15th Research Institute of China Electronics Technology Group Corporation, Beijing, China; 4 Department of Computer Science and Technology, Tsinghua University, Beijing, China; University of Wisconsin-Eau Claire, UNITED STATES

## Abstract

Gaze estimation plays a critical role in human-centered vision applications such as human–computer interaction and virtual reality. Although significant progress has been made in automatic gaze estimation by deep convolutional neural networks, it is still difficult to directly deploy deep learning based gaze estimation models across different edge devices, due to the high computational cost and various resource constraints. This work proposes LiteGaze, a deep learning framework to learn architectures for efficient gaze estimation via neural architecture search (NAS). Inspired by the once-for-all model (Cai et al., 2020), this work decouples the model training and architecture search into two different stages. In particular, a supernet is trained to support diverse architectural settings. Then specialized sub-networks are selected from the obtained supernet, given different efficiency constraints. Extensive experiments are performed on two gaze estimation datasets and demonstrate the superiority of the proposed method over previous works, advancing the real-time gaze estimation on edge devices.

## Introduction

Gaze estimation is a task that has gained increasing importance in recent years due to its potential to enhance human–computer interaction [1], virtual reality [2] and open dialogue system [3]. The goal of gaze estimation is to predict where a person is looking at given their face images [4]. This technique has been used for various applications such as eye tracking in virtual reality, sign language recognition, and gaze-based user interfaces.

One of the primary approaches for gaze estimation is the appearance-based method [5–7], where a direct nonlinear mapping is learned between images and gaze angles. Deep convolutional neural networks have shown significant improvements in the accuracy of appearance-based gaze estimation in recent years. A common practice is to adopt popular deep networks like VGGNet [8] and ResNet-50 [9] as backbones for feature extraction and then predict the gaze direction. However, existing methods mainly focus on improving the accuracy of gaze estimation, often ignoring computational efficiency. This approach makes it difficult to achieve real-time gaze estimation on edge devices, which have limited computational resources. Therefore, there is a need for the development of gaze estimation models that can maintain high accuracy while being computationally efficient.

In this paper, efficient deep convolutional architecture is investigated for gaze estimation. The proposed method takes inspiration from recent works on neural architecture search (NAS) [10, 11] and proposes a learning framework, *LiteGaze*, to learn efficient architectures for gaze estimation. Specifically, this work follows [10] to decouple the search procedure into the supernet training stage and sub-network search stage. In the first training stage, a supernet is designed to support diverse architectural settings, including network depth, width as well as kernel size. The training goal is to improve the accuracy of all sub-networks derived by sampling different parts of the supernet model. In the second architecture search stage, a subset of sub-networks is randomly selected to train an accuracy predictor, which can directly predict the accuracy for given architecture configurations. In the end, a distilled sub-network is selected with a predictor-guided architecture search algorithm, given resource constraints such as FLOPS. Finally, extensive experiments on GazeCapture [12] and ETH-XGaze [13] demonstrate the effectiveness of the proposed LiteGaze framework. Our method is able to achieve a much better trade-off between accuracy and computation than other efficient models.

Concretely, the contribution of this work can be summarized as follows:

- The paper introduces LiteGaze, a deep learning framework for efficient gaze estimation that utilizes neural architecture search (NAS) to learn specialized deep learning models.
- The proposed method enables efficient sampling of specialized sub-networks given resource constraints, providing flexible support for various architectures without requiring additional training.
- The effectiveness of the proposed approach is validated on two benchmark datasets, GazeCapture and ETH-XGaze, demonstrating the superiority of the LiteGaze framework over previous efficient models. It can significantly improve the efficiency of gaze estimation while maintaining high accuracy at the same time.

## Related work

Gaze estimation aims to estimate where a person is looking at from a face image. Early model-based works [14–16] rely on visual features extracted from eye images like pupil center and iris contours to estimate gaze directions. However, these models are based on human-crafted features designed under limited observations. More recently, appearance-based gaze estimation has become popular with the advancement of deep learning techniques. GazeNet [17, 18] is one of the first deep appearance-based gaze estimation model based on a 16-layer VGGNet [8]. They also provide the MPIIGaze dataset in real-world settings. iTracker network [12] proposes a robust eye tracking model by fusing eye images, full-face images and face grid information as inputs. Their model is learned end-to-end without using any hand-engineered features such as head pose or eye centers. Dilated-Net [7] adopts dilated convolutions to improve the gaze estimation accuracy by extracting higher resolution features. CA-Net [19] proposes a coarse-to-fine strategy to predict gaze direction from face images. Gaze360 [20] presents a large number of diverse annotated data for robust 3D gaze estimation in an unconstrained environment. It further proposes a 3D gaze model to extend existing models to include temporal information. AGE-Net [21] incorporates an attention mechanism to improve gaze estimation accuracy. Recently, L2CS-NET [22] proposes to improve the model generalization by simultaneously designing gaze classification and regression losses. Although these models have been proven effective in solving appearance-based gaze estimation problems, all these approaches are too computationally intensive to be deployed on edge devices. This work focuses on efficient gaze estimation models.

Previous research has attempted to solve constrained benchmark engineering optimization problems while maintaining a low computational cost [23–25]. In the context of deep learning, two commonly adopted approaches to accelerate deep convolutional neural networks include designing efficient architectures directly and optimizing network parameters through compression techniques. There are two commonly adopted approaches to accelerate deep convolutional neural networks. The first one is designing efficient architectures directly. MobileNet [26, 27] uses depthwise separable convolutions to build lightweight deep neural networks for mobile and embedded vision applications. ShuffleNet [28, 29] adopts pointwise group convolution and channel shuffle operations to reduce computation cost while maintaining accuracy. PVCNN [30] represents 3D input data in points to reduce the memory consumption. Another way for model acceleration is to compress the existing large models. Some works aim to prune the redundancy inside connections and convolution channels [31–33]. For example, Deep Compression [31] prunes the network by learning the most important connections. In addition, other works focus on weight quantization [34, 35]. Recently, Lemley et al. [36], propose a hardware-optimized network for efficient appearance-based gaze estimation. Oh et al. [37] propose to reduce the computational cost with convolution projection when applying self-attention operations. However, their method is manually designed and still faces significant performance degradation when computational constraints become stricter. By contrast, this work seeks to obtain efficient gaze estimation architectures through neural architecture search.

Neural architecture search (NAS) has been a popular research direction to automate the architecture design process [38–40]. The main idea of NAS is to search for the optimal neural network architecture, which can achieve a high level of performance on a given task, and has achieved great success on large-scale image classification tasks. It has been demonstrated that automatically searched deep models can outperform hand-crafted ones. Early NAS approaches [40, 41] mainly focus on high-precision architecture without considering the models’ efficiency. Recently, in order to improve the inference efficiency, other works [42, 43] try to incorporate the hardware constraints into architecture search. In addition, one-shot NAS methods [10, 11, 44, 45] are proposed to address the challenge of efficient inference across many devices and resource constraints. One-shot NAS aims to identify a single model that can perform well on different tasks and is suitable for different devices. The key idea is to enforce different sub-networks to share the same set of weights. Although one-shot NAS has been successful in various computer vision tasks, to the best of our knowledge, it has not been applied to gaze estimation. This work follows the once-for-all strategy [10] to search for efficient architectures for appearance-based gaze estimation. As a result, the proposed method is flexible and can support different architectural settings without requiring additional training.

## Method

### Overview

**Fig 1:**
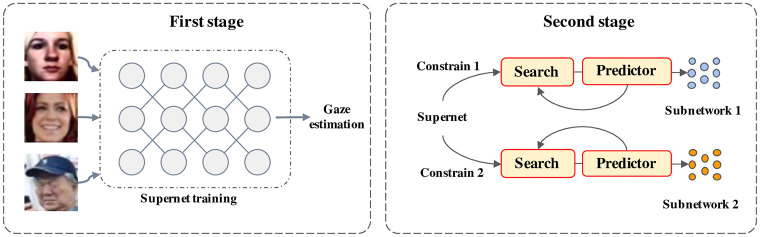
Overview of the proposed method. In the first stage, a supernet model is trained for gaze estimation. In the second stage, a subset of sub-networks is sampled and trained. During the test stage, specialized sub-networks are searched for given efficiency constraints.

The aim of this work is to achieve efficient gaze estimation via neural architecture search. To achieve this goal, a learning framework called *LiteGaze* is proposed by following once-for-all search strategy [10]. In particular, a supernet (largest) gaze estimation model is first trained and then specialized sub-networks are derived for given deployment constraints. As a result, the proposed method is able to flexibly support different architectures without additional training. Fig 1 shows the overall flow of the pipeline of the proposed method and the details are provided in the following subsections. The training and search procedure are summarized in Algorithm 1 and 2.

### One-shot supernet training

In the first stage, a gaze estimation supernet is trained and it supports many sub-networks of different sizes. During the training, three important configurations of convolutional neural networks are considered, i.e., depth, width (number of channels) and convolutional kernel size. To ensure accuracy and training efficiency, progressive shrinking [10] is adopted to perform a progressive training sequence from large models to small models by controlling network configurations. Note that all the sub-networks share the parameters with the supernet.

#### Architecture

This work’s model follows the architecture space of MobileNetV3. [46]. In particular, the convolutional kernel size can be chosen from {3, 5, 7}, the depth of one unit can be chosen from {2, 3, 4}, and the width expansion ratio can be chosen from {3, 4, 6}. By selecting different configuration combinations, plenty of sub-networks with different architectures can be randomly sampled via weight sharing. In this way, each sub-network can be trained and operate independently.

#### Progressive shrinking

Since there are too many sub-networks that can be sampled from the supernet and small sub-networks are nested in large sub-networks, the progressive shrinking [10] is employed to dynamically train the supernet from large models to small ones. Specifically, the largest gaze estimation model is first trained with maximal kernel size (7 × 7), depth (4) and width expansion ratio (6).

**Fig 2:**
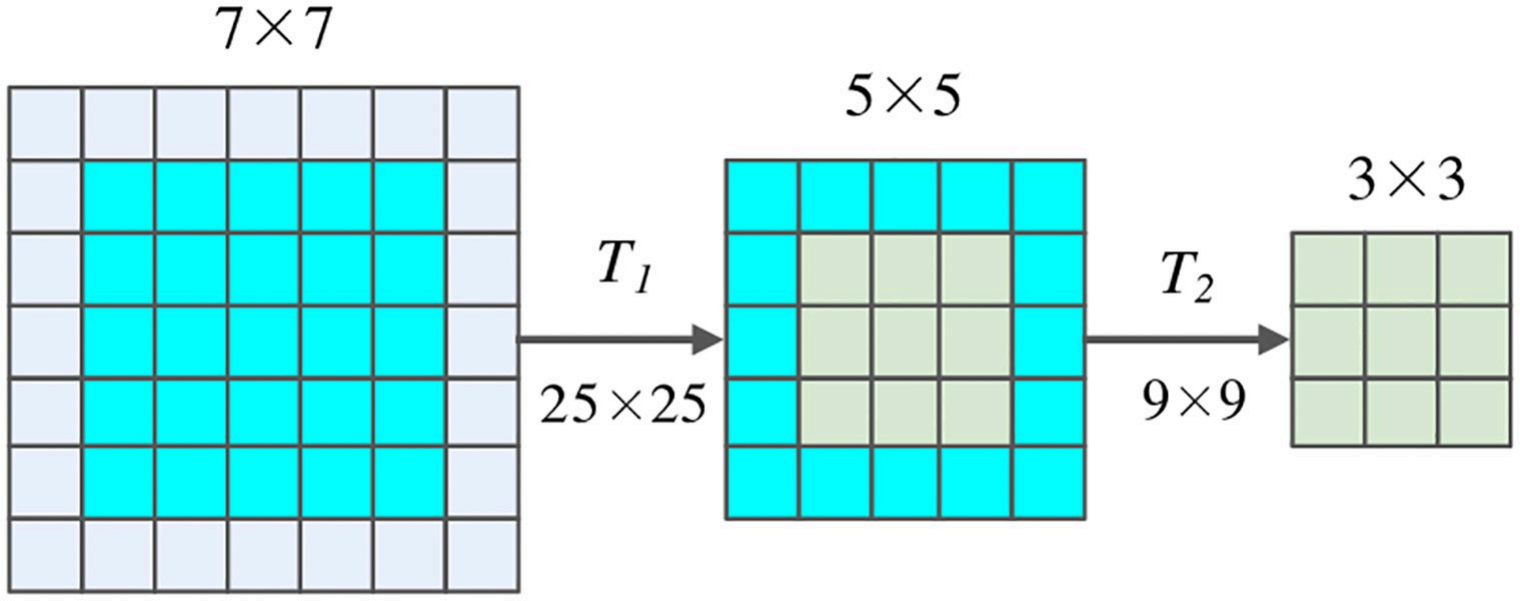
Illustration of kernel size shrinking. The center of the larger kernel is reused as the smaller kernel. In addition, kernel transformation is further performed used when sharing the kernel weights.

For kernel size shrinking (as illustrated in Fig 2), the center of the 7 × 7 convolutional kernel is reused as a 5 × 5 kernel, and the center of which can be used as a 3 × 3 kernel. To improve the flexibility of sub-networks, kernel transformation is further performed when sharing the kernel weights. In this way, the small-sized convolution kernel can be obtained by performing matrix multiplication with the parameters of the larger-sized convolution kernel through the transformation matrix (*T*_1_ and *T*_2_). During the experiments, different transformation matrices are used in different layers and the same matrix are shared among different channels in the same layer.

For depth shrinking, various models with *n* layers (the total number of layers of the supernet) are first trained. Then the sub-networks are sampled by only retaining the first *d* layers and ignoring the rest *n* − *d* layers. In this way, the weights of the first *d* layers of small models can be initialized with larger models.

For width shrinking, a full-width model is first trained by using all the channels. Then the targeted channels are sampled by using a ranking strategy, based on the importance of each channel. Specifically, the *L*_1_ norm of a channel’s weight is calculated and the higher values are considered to have higher importance. Thus, the sub-networks with shrunken widths are initialized with the selected channels in larger models.

**Algorithm 1** Stage 1: Supernet Training

 **Require:** *SuperNet* with parameters *W*, *SubNet* with parameters *W*_*conf*_ given a configuration *conf*(*d*, *w*, *k*) given depth *d*, width *w* and kernel size *k*.

 **repeat**

  *x*, *y* ← random mini- batch data from the dataset

  *z* ← *SuperNet*(*x*;*W*)

  $L_{sup}\leftarrow {∥z-y∥}_{1}$

  $W\overset{+}{\leftarrow }-\nabla_{W}L_{sup}$

 **until** convergence of parameters

 **repeat**

  *conf*(*d*_*i*_, *w*_*i*_, *k*_*i*_) ← get network configuration

  *SubNet* with weight *W*_*conf*_ ← *conf* *x*, *y* ← random mini- batch from the dataset

  *z* ← *SubNet*(*x*;*W*_*conf*_)

  $L_{sub}={∥z-y∥}_{1}$ $W_{conf}\overset{+}{\leftarrow }-\nabla_{W_{conf}}L_{sub}$

 **until** convergence of parameters

**Algorithm 2** Stage 2: Sub-network Search

 **Require:** Accuracy Predictor *APNet* with weight *W*_*ap*_, FLOPS constraint *FLOPS*_*max*_, Pretrained *SuperNet*.

 **repeat**

  *conf*(*d*_*i*_, *w*_*i*_, *k*_*i*_) ← get network configuration

  *SubNet* with weight *W*_*conf*_ ← *conf*, *SuperNet*

  *x*, *y* ← random mini- batch from the dataset

  *z* ← *SubNet*(*x*;*W*_*conf*_)

  *error*_*data*_ ← ‖*z* − *y*‖_1_*error*_*conf*_ ← *APNet*(*conf*)

  $L_{ap}={∥{error}_{data}-{error}_{conf}∥}_{2}$

  $W_{ap}\overset{+}{\leftarrow }-\nabla_{W_{ap}}L_{ap}$

 **until** convergence of parameters

 Use evolutionary search based on *APNet* to get the optimal architecture given *FLOPS*_*max*_.

#### Training objective

The weights of the supernet are denoted as *W*, and a sampled sub-network is denoted as *SubNet*, defined by *conf*_*i*_, which represents different architectural configurations using network depth, width, and kernel size. The training objective is to optimize *W* to achieve the best average precision for all sub-networks and the supernet. The training objective can be formulated as:

$$\begin{array}{c} \underset{W}{\text{min}}\sum_{conf_{i}}{∥SubNet\left(x;W,conf_{i}\right)-y∥}_{1} \end{array}$$ (1)

where *x*, *y* are the input face image and the corresponding label.

### Sub-network search

Once the supernet has been properly trained, specific sub-networks that meet the efficiency constraints can be identified through a search process. Since the supernet is trained with weight sharing, the obtained sub-networks can be directly evaluated without further finetuning. The network search consists of two steps. First, an accuracy predictor is trained to predict the accuracy for a given architecture setting. Then a predictor-guided architecture search algorithm is utilized to select a distilled sub-network that meets the specified target constraints.

#### Accuracy predictor

**Fig 3:**
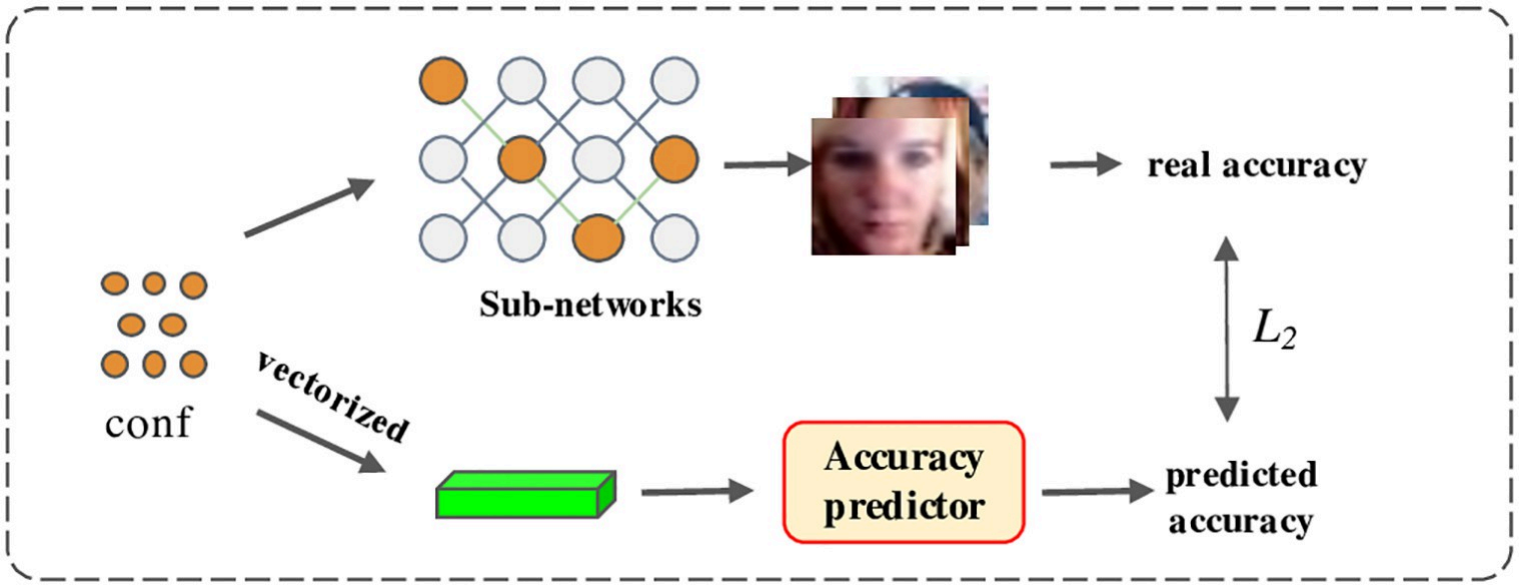
The training framework of the accuracy predictor. The proposed method randomly samples a large number of sub-networks with different configurations and measures their corresponding accuracy. An accuracy predictor is then trained to estimate the accuracy of each sub-network directly from its configuration.

The accuracy predictor is used to estimate the accuracy directly from the sub-network configurations. The overall framework is shown in Fig 3. In particular, the accuracy predictor is a 3-layer multi-layer perceptron (MLP) with 256 hidden units, and the ReLU layer is used as the activation function. The sub-network configurations (i.e, kernel size, depth, and width) are encoded as one-hot vectors, which are then concatenated and fed to the MLP model. The output is the estimated accuracy for the given configuration. To construct the training data, a large number of sub-networks are randomly sampled, and the estimated accuracy from the face images is collected. Then the mean square error (MSE) between the collected accuracy and that predicted by the accuracy predictor is calculated and used as the loss function. As a result, the well-trained accuracy predictor is able to approximate the final performance of the sub-network by only using the corresponding configurations.

#### Architecture search

Since the accuracy predictor can provide quick feedback on the performance of models, the evolutionary search [40] is used based on the accuracy predictor to get the optimal architecture for target efficiency constraints (e.g. FLOPS). After finding optimal configurations, the corresponding sub-networks are further finetuned for several epochs to improve the performance further.

## Experiment

### Experimental settings

#### Datasets

The experiments are conducted using two datasets, namely GazeCapture [12] (https://gazecapture.csail.mit.edu/) and ETH-XGaze [13] (https://ait.ethz.ch/projects/2020/ETH-XGaze/). GazeCapture is a large-scale gaze estimation dataset, containing 2,445,504 images with over 1,450 people. This dataset is collected from mobile devices with variable lighting conditions and unconstrained head motion. ETH-XGaze [13] consists of more than 1 million high-resolution images for gaze estimation. It covers large head poses and gaze ranges from 110 subjects of different ages, genders and races with consistent label quality.

#### Training details

In the experiment, the images are resized to 128 × 128. The Adam solver is utilized with a batch size of 64 to optimize the supernet. The initial learning rate is 1 × 10^−3^ and a cosine annealing schedule is employed to decrease the learning rate. When training the sub-networks, the initial learning rate is set to 1 × 10^−4^. For sub-network search, FLOPS (floating point operations per second) is used as the efficiency constraint to measure how many operations are needed to run the model. An evolutionary algorithm is used to find the optimal sub-networks given specific FLOPS. The experiments are implemented with PyTorch on one Tesla V100 32GB GPU. In addition, we keep the hyperparameters and other training settings consistent with the once-for-all work [10].

### Results

In the experiments, FLOPS is used as the efficiency constraint. The largest supernet has 186.98M FLOPS and the smallest sub-network has 40.20M FLOPS. An evolutionary search is employed to automatically find different deep architectures given different FLOPS budgets.

#### Accuracy predictor evaluation

**Fig 4:**
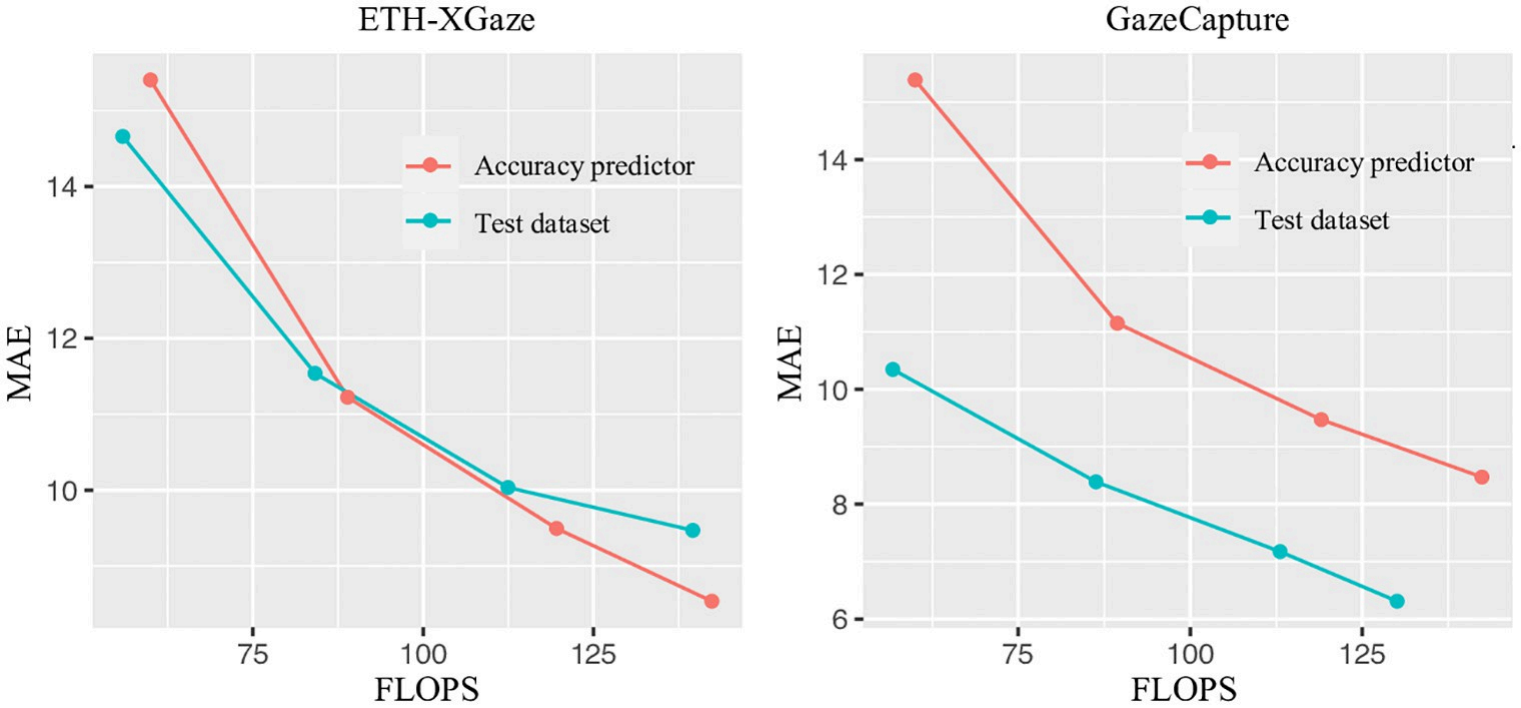
Evaluation for accuracy predictor on ETH-XGaze and GazeCapture datasets.

The effectiveness of the accuracy predictor is first evaluated. When searching for specialized sub-networks, the search constraints for FLOPS are set at 60M, 90M, 120M, and 150M, respectively. Fig 4 shows the results evaluated on ETH-XGaze and GazeCapture datasets. The *x*-axis denotes the FLOPS of the sub-networks, and the *y*-axis is the corresponding Mean Angular Error (MAE) values [20]. It estimates the angles between the predicted gaze vectors and ground truth. In particular, comparing the results of the accuracy predictor (red line) with those evaluated on test images (cyan line), a similar decreasing trend in MAE values is observed as more FLOPS are allowed. These results validate the effectiveness of the accuracy predictor in predicting the accuracy of a model given its architecture configurations. It can be noticed that the difference between the red and cyan lines is larger for the GazeCapture dataset compared to the ETH-XGaze dataset. This could be attributed to the fact that the GazeCapture dataset includes a larger variety of unconstrained conditions and diverse settings, making it a more challenging dataset for gaze estimation.

#### Comparing with other methods

This part presents a comprehensive comparison of the proposed method with state-of-the-art efficient models for gaze estimation on the ETH-XGaze and GazeCapture datasets. The results of four models, arranged from smallest to largest based on different FLOPS, are presented. In particular, LiteGaze-XS, LiteGaze-S, LiteGaze-M and LiteGaze-L are the models sampled by setting the search constraint of FLOPS to 60M, 90M, 120M and 150M, respectively. The compared models are implemented with the widely used Timm library [47]. The MAE and FLOPS for each model are summarized in Table 1. As can be seen, the method presented in this work consistently outperforms other efficient methods (such as MobileNet, EfficientNet and TinyNet), while reducing many orders of magnitude computations. For instance, the LiteGaze-S model achieves 11.54 and 8.39 MAE on the two datasets, respectively, with less than 90M FLOPS. By contrast, MobileNetV2 can only achieve 19.76 and 9.14 MAE with similar FLOPS. Moreover, the performance can be further improved by fine-tuning the obtained sub-networks (as denoted with *). The results show that the smallest model, LiteGaze-XS, achieved 3.71 MAE on the GazeCapture dataset, outperforming ResNet18 that requires significantly more computations.

**Table 1:** Comparison with SOTA efficient models on ETH-XGaze and GazeCapture datasets. The Mean Angular Error (MAE) is used to evaluate the gaze estimation performance of different models, along with the corresponding FLOPS.

Model	ETH-XGaze		GazeCapture	
	MAE	FLOPS	MAE	FLOPS
Resnet18	7.867	592.18M	4.21	592.18M
EfficientNet	20.48	125.36M	4.37	125.36M
MobileNetV2	19.76	97.79M	9.14	97.79M
TinyNet	12.16	90.01M	7.27	90.01M
LiteGaze-XS	14.66	55.88M	10.35	56.76M
LiteGaze-S	11.54	84.12M	8.39	86.29M
LiteGaze-M	10.03	112.45M	7.17	113.05M
LiteGaze-L	9.47	139.59M	6.31	130.06M
LiteGaze-XS*	6.93	55.88M	3.71	56.76M
LiteGaze-S*	6.82	84.12M	4.05	86.29M
LiteGaze-M*	7.83	112.45M	4.76	113.05M
LiteGaze-L*	7.63	139.59M	3.82	130.06M

The * represents the results obtained by fine-tuning the specialized sub-network.

Additionally, a Kruskal-Wallis test, a non-parametric alternative to One-Way ANOVA is conducted, for further analysis. In particular, the results are divided into three groups as shown in Table 1. The resulting statistics and p-values for ETH-XGaze and GazeCapture are (7.73, 0.02) and (6.27, 0.04), respectively. Since both p-values are less than 0.05, our proposed method is statistically significantly better than the compared methods.

These results validate the effectiveness of the proposed approach and highlight the importance of efficient gaze estimation models, especially for edge devices with limited resources. Moreover, unlike previous gaze estimation approaches that require additional training to support different architectures, the proposed method enables the quick sampling of specialized sub-networks given resource constraints. This flexibility makes LiteGaze suitable for various applications with diverse requirements.

## Conclusion

This study presents LiteGaze, an efficient deep learning model for human gaze estimation that utilizes neural architecture search (NAS) to discover specialized architectures with efficiency constraints. The proposed supernet can support various architectural settings including network depth, width, and kernel size, and specialized sub-networks can be quickly sampled without additional training. The results from extensive experiments on ETH-XGaze and GazeCapture datasets demonstrate that the proposed method can improve the trade-off between accuracy and computations compared to previous methods, making it a valuable contribution to the field of real-time gaze estimation. However, a main limitation of this approach is that training different architectures can require additional resources and time during the training stage, which may make it more time-consuming and resource-intensive compared to some other methods.

## References

[1] Fridman L, Reimer B, Mehler B, Freeman WT. Cognitive Load Estimation in the Wild. In: Proc. CHI Conference on Human Factors in Computing Systemsn; 2018. p. 1–9.

[2] Patney A, Kim J, Salvi M, Kaplanyan A, Wyman C, Benty N, et al. Perceptually-based foveated virtual reality. In: ACM SIGGRAPH 2016 emerging technologies; 2016. p. 1–2.

[3] Li L, Yu X, Li J, Wang G, Shi JY, Tan YK, et al. Vision-based attention estimation and selection for social robot to perform natural interaction in the open world. In: ACM/IEEE International Conference on Human-Robot Interaction. IEEE; 2012. p. 183–184.

[4] Guo T, Liu Y, Zhang H, Liu X, Kwak Y, Yoo B, et al. A Generalized and Robust Method Towards Practical Gaze Estimation on Smart Phone. In: Proc. Int. Conf. on Computer Vision; 2019. p. 1131–1139.

[5] D MLR, Biswas P. Appearance-Based Gaze Estimation Using Attention and Difference Mechanism. In: Proceedings of the IEEE/CVF Conference on Computer Vision and Pattern Recognition (CVPR) Workshops; 2021. p. 3143–3152.

[6] Cheng Y, Huang S, Wang F, Qian C, Lu F. A Coarse-to-Fine Adaptive Network for Appearance-Based Gaze Estimation. In: Proc. AAAI Conf. on Artificial Intelligence. AAAI Press; 2020. p. 10623–10630.

[7] Chen Z, Shi BE. Appearance-based gaze estimation using dilated-convolutions. In: Proc. Asia Conf. on Computer Vision. Springer; 2018. p. 309–324.

[8] Simonyan K, Zisserman A. Very Deep Convolutional Networks for Large-Scale Image Recognition. In: Proc. Int. Conf. on Learning Representations; 2015.

[9] He K, Zhang X, Ren S, Sun J. Deep residual learning for image recognition. In: Proceedings of the IEEE conference on computer vision and pattern recognition; 2016. p. 770–778.

[10] Cai H, Gan C, Wang T, Zhang Z, Han S. Once-for-All: Train One Network and Specialize it for Efficient Deployment. In: Proc. Int. Conf. on Learning Representations; 2020.

[11] Liu H, Simonyan K, Yang Y. DARTS: Differentiable Architecture Search. In: Proc. Int. Conf. on Learning Representations; 2018.

[12] Krafka K, Khosla A, Kellnhofer P, Kannan H, Bhandarkar S, Matusik W, et al. Eye tracking for everyone. In: Proc. IEEE Conf. on Computer Vision & Pattern Recognition; 2016. p. 2176–2184.

[13] Zhang X, Park S, Beeler T, Bradley D, Tang S, Hilliges O. Eth-xgaze: A large scale dataset for gaze estimation under extreme head pose and gaze variation. In: Proc. Euro. Conf. on Computer Vision; 2020. p. 365–381.

[14] Borgestig M, Sandqvist J, Ahlsten G, Falkmer T, Hemmingsson H. Gaze-based assistive technology in daily activities in children with severe physical impairments-An intervention study. Developmental Neurorehabilitation. 2017;20(3):129–141. doi: 10.3109/17518423.2015.1132281 26930111

[15] Yamazoe H, Utsumi A, Yonezawa T, Abe S. Remote gaze estimation with a single camera based on facial-feature tracking without special calibration actions. In: Proceedings of the 2008 symposium on Eye tracking research & applications; 2008. p. 245–250.

[16] Valenti R, Sebe N, Gevers T. Combining head pose and eye location information for gaze estimation. IEEE Transactions on Image Processing. 2011;21(2):802–815. doi: 10.1109/TIP.2011.2162740 21788191

[17] Zhang X, Sugano Y, Fritz M, Bulling A. Mpiigaze: Real-world dataset and deep appearance-based gaze estimation. IEEE Trans Pattern Analysis & Machine Intelligence. 2017;41(1):162–175. doi: 10.1109/TPAMI.2017.2778103 29990057

[18] Zhang X, Sugano Y, Fritz M, Bulling A. Appearance-based gaze estimation in the wild. In: Proc. IEEE Conf. on Computer Vision & Pattern Recognition; 2015. p. 4511-4520.

[19] Cheng Y, Huang S, Wang F, Qian C, Lu F. A coarse-to-fine adaptive network for appearance-based gaze estimation. In: Proc. AAAI Conf. on Artificial Intelligence. vol. 34; 2020. p. 10623-10630.

[20] Kellnhofer P, Recasens A, Stent S, Matusik W, Torralba A. Gaze360: Physically unconstrained gaze estimation in the wild. In: Proc. Int. Conf. on Computer Vision; 2019. p. 6912-6921.

[21] Biswas P, et al. Appearance-based gaze estimation using attention and difference mechanism. In: Proc. IEEE Conf. on Computer Vision & Pattern Recognition; 2021. p. 3143-3152.

[22] Abdelrahman AA, Hempel T, Khalifa A, Al-Hamadi A. L2CS-Net: Fine-Grained Gaze Estimation in Unconstrained Environments. arXiv preprint arXiv:220303339. 2022;.

[23] Agushaka JO, Ezugwu AE, Olaide ON, Akinola O, Zitar RA, Abualigah L. Improved Dwarf Mongoose Optimization for Constrained Engineering Design Problems. Journal of Bionic Engineering. 2022; p. 1–33. doi: 10.1007/s42235-022-00316-8 36530517 PMC9745293

[24] Agushaka JO, Akinola O, Ezugwu AE, Oyelade ON, Saha AK. Advanced dwarf mongoose optimization for solving CEC 2011 and CEC 2017 benchmark problems. Plos one. 2022;17(11):e0275346. doi: 10.1371/journal.pone.0275346 36322574 PMC9629639

[25] Agushaka JO, Ezugwu AE, Abualigah L. Gazelle Optimization Algorithm: A novel nature-inspired metaheuristic optimizer. Neural Computing and Applications. 2022; p. 1–33. 10.1007/s00521-022-07705-4 PMC9424068 36060097

[26] Howard AG, Zhu M, Chen B, Kalenichenko D, Wang W, Weyand T, et al. Mobilenets: Efficient convolutional neural networks for mobile vision applications. arXiv preprint arXiv:170404861. 2017;.

[27] Sandler M, Howard A, Zhu M, Zhmoginov A, Chen LC. Mobilenetv2: Inverted residuals and linear bottlenecks. In: Proc. IEEE Conf. on Computer Vision & Pattern Recognition; 2018. p. 4510-4520.

[28] Zhang X, Zhou X, Lin M, Sun J. Shufflenet: An extremely efficient convolutional neural network for mobile devices. In: Proceedings of the IEEE conference on computer vision and pattern recognition; 2018. p. 6848-6856.

[29] Ma N, Zhang X, Zheng HT, Sun J. Shufflenet v2: Practical guidelines for efficient cnn architecture design. In: Proc. Euro. Conf. on Computer Vision; 2018. p. 116-131.

[30] Liu Z, Tang H, Lin Y, Han S. Point-voxel cnn for efficient 3d deep learning. Proc Conf on Neural Information Processing Systems. 2019;32.

[31] Han S, Mao H, Dally WJ. Deep compression: Compressing deep neural networks with pruning, trained quantization and huffman coding. arXiv preprint arXiv:151000149. 2015;.

[32] He Y, Zhang X, Sun J. Channel pruning for accelerating very deep neural networks. In: Proceedings of the IEEE international conference on computer vision; 2017. p. 1389–1397.

[33] Wen W, Wu C, Wang Y, Chen Y, Li H. Learning structured sparsity in deep neural networks. Proc Conf on Neural Information Processing Systems. 2016;29.

[34] Courbariaux M, Hubara I, Soudry D, El-Yaniv R, Bengio Y. Binarized neural networks: Training deep neural networks with weights and activations constrained to+ 1 or-1. arXiv preprint arXiv:160202830. 2016;.

[35] Wang K, Liu Z, Lin Y, Lin J, Han S. Haq: Hardware-aware automated quantization with mixed precision. In: Proc. IEEE Conf. on Computer Vision & Pattern Recognition; 2019. p. 8612-8620.

[36] Lemley J, Kar A, Drimbarean A, Corcoran P. Efficient CNN implementation for eye-gaze estimation on low-power/low-quality consumer imaging systems. arXiv preprint arXiv:180610890. 2018;.

[37] O Oh J, Chang HJ, Choi SI. Self-Attention With Convolution and Deconvolution for Efficient Eye Gaze Estimation From a Full Face Image. In: Proc. IEEE Conf. on Computer Vision & Pattern Recognition; 2022. p. 4992-5000.

[38] Cai H, Chen T, Zhang W, Yu Y, Wang J. Efficient architecture search by network transformation. In: Proc. AAAI Conf. on Artificial Intelligence. vol. 32; 2018.

[39] Zoph B, Le QV. Neural Architecture Search with Reinforcement Learning. In: Proc. Int. Conf. on Learning Representations; 2017.

[40] Real E, Aggarwal A, Huang Y, Le QV. Regularized evolution for image classifier architecture search. In: Proc. AAAI Conf. on Artificial Intelligence. vol. 33; 2019. p. 4780-4789.

[41] Zoph B, Vasudevan V, Shlens J, Le QV. Learning transferable architectures for scalable image recognition. In: Proc. IEEE Conf. on Computer Vision & Pattern Recognition; 2018. p. 8697-8710.

[42] Cai H, Zhu L, Han S. ProxylessNAS: Direct Neural Architecture Search on Target Task and Hardware. In: Proc. Int. Conf. on Learning Representations; 2019.

[43] Tan M, Chen B, Pang R, Vasudevan V, Sandler M, Howard A, et al. Mnasnet: Platform-aware neural architecture search for mobile. In: Proc. IEEE Conf. on Computer Vision & Pattern Recognition; 2019. p. 2820-2828.

[44] Bender G, Kindermans PJ, Zoph B, Vasudevan V, Le Q. Understanding and simplifying one-shot architecture search. In: Proc. Int. Conf. on Machine Learning. PMLR; 2018. p. 550-559.

[45] Guo Z, Zhang X, Mu H, Heng W, Liu Z, Wei Y, et al. Single path one-shot neural architecture search with uniform sampling. In: Proc. Euro. Conf. on Computer Vision; 2020. p. 544-560.

[46] Howard A, Sandler M, Chu G, Chen LC, Chen B, Tan M, et al. Searching for mobilenetv3. In: Proc. Int. Conf. on Computer Vision; 2019. p. 1314-1324.

[47] Wightman R. PyTorch Image Models; 2019. https://github.com/rwightman/pytorch-image-models.

